# Some Forms of Dyspepsia

**Published:** 1877-01-20

**Authors:** 


### SOME FORMS OF DYSPEPSIA.

Dr. F. Delafield (*Amer. Clinical Lectures,* Vol. 2, No. 4,) under the above title gives some valuable suggestions: (1.) Dyspepsia confined to the stomach has the following symptoms, viz.: attacks of pain and vomiting, coming at first at long and then at short intervals—the attack always excited by the ingestion of food and the pain ceasing when the stomach is emptied. The disease lasts for years, and steadily grows worse. Medical treatment alleviates the symptoms for longer or shorter intervals, but never permanently.

The most rational and effectual treatment of these cases is the systematic use of the stomach-pump. The pump, as a rule, is not to be used till three hours after a meal of solid food. The patient soon learns to use the pump at home. (2.) Dyspepsia due to functional derangement of the small intestine, the stomach being unaffected. Symptoms—pain is the most troublesome and may be referred to any part of the abdominal cavity. It is usually described as a constant dull pain—has no special relation to the ingestion of food or its quality. It occurs when the stomach is full or empty—whether the food is spare and simple or abundant and rich. The use of liquor will usually stop it for a short time. There may be some particular time of the day at which the pain comes on with tolerable regularity. There may be nausea, but not vomiting. Appetite is often good, and food causes no distress. Some cases are easily relieved by treatment; others prove obstinate. Drugs indicated are, cubebs, ipecac, and assafœtida. Horseback riding is often of great service.

(3.) Dyspepsia from disordered function of the liver. Clinically, these cases can be divided into two classes: (*a.*) Those of florid complexion and of well-developed adipose and muscular tissues. (*b.*) Those of pallid complexion, spare figure and feeble muscles. In the first class the symptoms are due to derangement of those functions of the liver which should effect the destructive metamorphosis of albuminoid substances, so that patients receive a full supply of the nutritious portions of the food, but do not get rid of the excrementitious. In the second class, the functions which should assimilate the fat and peptones are so disordered that the patient is imperfectly nourished. In one case the tissues are overmanured, but badly drained; in the other, they are well enough drained but not manured at all.

*Symptoms* of the first class of cases are—depression of spirits, liability to attacks of vertigo, bowels more or less irregular, urine apt to contain an excess of uric acid or of the urates, partial loss of memory, an inability to apply the mind to business. Treatment—an entire abstinence from every kind of alcoholic drink; also from tobacco; vigorous outdoor muscular exercise; drugs as indicated.

Symptoms of the second class of cases — flatulence, headache, curious nervous feelings in various parts of the body, sleeplessness, hypochondria often, irregular action of the heart, pain in the precordial region and a dull pain in the right hypochondriac region, extending to the back and shoulder, constipation, emaciation, urine normal usually. Treatment of this condition is different. Diet must be carefully regulated—full and nutritious; wines, etc., are often of service; cream and cod liver oil are sometimes indicated; constipation must be relieved, nervous symptoms allayed, appetite improved by the mineral acids, exercising in the open air, bathing the entire body daily in cold water.

